# Aberrant brain dynamics and spectral power in children with ADHD and its subtypes

**DOI:** 10.1007/s00787-022-02068-6

**Published:** 2022-08-22

**Authors:** Na Luo, Xiangsheng Luo, Suli Zheng, Dongren Yao, Min Zhao, Yue Cui, Yu Zhu, Vince D. Calhoun, Li Sun, Jing Sui

**Affiliations:** 1 Institute of Automation, Chinese Academy of Sciences, Brainnetome Center and National Laboratory of Pattern Recognition, Beijing, 100190 China; 2 University of Chinese Academy of Sciences, Beijing, 100049 China; 3 Peking University Sixth Hospital and, Peking University Institute of Mental Health, Beijing, 100191 China; 4 NHC Key Laboratory of Mental Health (Peking University) and National Clinical Research Center for Mental Disorders, (Peking University Sixth Hospital), Beijing, 100191 China; 5 Massachusetts Eye and Ear Infirmary, Boston, MA 02114 USA; 6 Harvard Medical School, Boston, MA 02114 USA; 7 Tri-Institutional Center for Translational Research in Neuroimaging and Data Science (TReNDS), Georgia State University, Georgia Institute of Technology, and Emory University, Atlanta, GA 30303 USA; 8 State Key Laboratory of Cognitive Neuroscience and Learning, Beijing Normal University, Beijing, 100875 China

**Keywords:** ADHD, Subtypes, EEG, Microstate dynamics, Spectral power

## Abstract

Attention-deficit/hyperactivity disorder (ADHD) is a prevalent neurodevelopmental disorder in children, usually categorized as three subtypes, predominant inattention (ADHD-I), predominant hyperactivity-impulsivity (ADHD-HI), and a combined subtype (ADHD-C). Yet, common and unique abnormalities of electroencephalogram (EEG) across different subtypes remain poorly understood. Here, we leveraged microstate characteristics and power features to investigate temporal and frequency abnormalities in ADHD and its subtypes using high-density EEG on 161 participants (54 ADHD-Is and 53 ADHD-Cs and 54 healthy controls). Four EEG microstates were identified. The coverage of salience network (state C) were decreased in ADHD compared to HC (*p* = 1.46e-3), while the duration and contribution of frontal–parietal network (state D) were increased (*p* = 1.57e-3; *p* = 1.26e-4). Frequency power analysis also indicated that higher delta power in the fronto-central area (*p* = 6.75e-4) and higher power of theta/beta ratio in the bilateral fronto-temporal area (*p* = 3.05e-3) were observed in ADHD. By contrast, remarkable subtype differences were found primarily on the visual network (state B), of which ADHD-C have higher occurrence and coverage than ADHD-I (*p* = 9.35e-5; *p* = 1.51e-8), suggesting that children with ADHD-C might exhibit impulsivity of opening their eyes in an eye-closed experiment, leading to hyper-activated visual network. Moreover, the top discriminative features selected from support vector machine model with recursive feature elimination (SVM-RFE) well replicated the above results, which achieved an accuracy of 72.7% and 73.8% separately in classifying ADHD and two subtypes. To conclude, this study highlights EEG microstate dynamics and frequency features may serve as sensitive measurements to detect the subtle differences in ADHD and its subtypes, providing a new window for better diagnosis of ADHD.

**Supplementary Information:**

The online version contains supplementary material available at 10.1007/s00787-022-02068-6.

## Introduction

Attention-deficit/hyperactivity disorder (ADHD), characterized by age-inappropriate inattention, hyperactivity and impulsivity, is a common neurodevelopmental disorder in children with an estimated prevalence about 5–6% [1]. It leaves a negative impact on children’s academic performance and social functions. According to the clinical manifestations, ADHD can be characterized by predominantly inattention (ADHD-I), predominant hyperactivity-impulsivity (ADHD-HI), and a combination of both (ADHD-C) in DSM-IV. Although subtypes reflect only the current symptom profile and are described as “presentations” in the DSM-5, differences in symptoms, function and brain function across dimensions suggest that commonalities and differences between subtypes are potentially valuable in understanding the pathogenesis of ADHD [2, 3].

Electroencephalogram (EEG) is readily accessible and inexpensive, which measures scalp electrical activity with millisecond temporal resolution produced by neuronal ensembles of the cerebral cortex [4]. It measures cortical electrical activity with high temporal resolution, while its poor spatial resolution precludes precise anatomical identification of underlying neural sources. The development of high-density EEG technology in recent years has, therefore, improved the spatial localization and resolution of EEG signals [5, 6], namely the 128 channels EEG acquisition system used in the current study. The temporally synchronized neural activity measured by EEG present reliable associations between frequency-specific oscillations and various cognitive functions, as well as their implication in various mental disorders [7, 8]. Resting-state EEG signals can be decomposed into waves that oscillate at different frequencies. Each sub-band occupies a specific portion of the spectrum and has its own characteristics [9]. The power in a particular frequency band can be expressed in absolute or relative terms through the Fast Fourier Transform (FFT) [10], which may quantify information about rhythms of the brain and provide possible neural marker for ADHD [11]. As mentioned in previous studies, the most discussed EEG feature is increased theta/beta ratio (TBR) [12], which is characterized as ratio of elevated power of slow waves (theta band) and decreased power of fast wave (beta wave). Compared to healthy controls (HC), ADHD-C subtype was characterized with markedly increased TBR with a widespread decrease in beta power, while ADHD-I subtype presented increased TBR with a global increase in theta power [13]. However, more recent studies have failed to replicate TBR differences in ADHD versus non-ADHD [14]. Thus, researchers still struggle to identify stable and sensitive frequency biomarkers for ADHD and its subtypes as well.

In parallel, microstates are global patterns of scalp potential topographies that remain quasi-stable for around 60–120 ms before changing to another quasi-stable map, which is considered to be the cornerstones of the mental states shown in EEG data [15, 16]. Studies showed that microstate analysis can help reveal the importance of the modularity of brain dynamics and their function in behavioral control and brain disease [17, 18]. Koening et al. presented four normative microstate maps for resting-state EEG data with a database of 496 subjects between the age of 6 and 80 years [16], which are highly reproducible and widely used in various pioneering work [19, 20]. Britz et al. extended to explore the relationship between the rapidly fluctuating EEG-defined microstates and the slowly oscillating fMRI-defined resting states, which indicated that the typical four EEG topographies were spatially correlated with four of the resting-state networks (RSN) located in bilateral superior and middle temporal gyri (RSN1), bilateral inferior occipital (RSN2), salience network (RSN3), and frontal-parietal network (RSN4) by general linear model (GLM) and independent component analysis (ICA) decomposition[21]. Moreover, it has been widely used to evaluate temporal abnormalities in schizophrenia [20, 22]. However, few studies have used microstates to investigate the abnormal temporal dynamics in children with ADHD and their subtypes. One example is from Cevallos et al. [23], which emphasized the ADHD-HC differences using the global field power (GFP), while more work on ADHD subtypes discrimination is still to be carried out.

In this study, we leverage microstate characteristics and power features to investigate temporal and frequency abnormalities in ADHD and its subtypes using high-density EEG. To achieve these goals, we first calculated four microstate features on ADHD and subtypes, following with group differences comparison for each microstate parameter through a variety of statistical methods. Then independent component analysis (ICA) was conducted on the absolute power of each frequency band to extract coherent electroencephalogram variations within several-related channels across subjects, which were further compared between groups. Finally, a support vector machines model with recursive feature elimination (SVM-RFE) algorithm was adopted to investigate temporal and frequency features with high discriminative power.

## Materials and methods

### Participants

A total of 161 participants, 8–15 years of age, were recruited in the study (123 males, 38 females). The children with ADHD (*n* = 107) were enrolled from the Peking University Sixth Hospital in Beijing. 54 HCs matched for sex and age were recruited from communities in Beijing. Written informed consent was obtained from all the children and their parents. The study was approved by the Ethics Committee of Peking University Sixth Hospital/Institute of Mental Health. All subjects were interviewed and underwent diagnosis to ADHD using DSM-IV criterion by a qualified psychiatrist. The Kiddie Schedule for Affective Disorders and Schizophrenia for School-Age Children (K-SADS) was used to confirm the diagnosis and subtypes in the ADHD group. Considering the little sample size of hyperactivity subtype, only inattentive subtype (ADHD-I, *n* = 54) and combined subtype (ADHD-C, *n* = 53) were included in the present study. All subjects were first diagnosed patients in the clinics and did not receive stable intervention and treatment at present. All the participants met the following criteria: (a) no history of head trauma with a loss of consciousness, neurological illness or other severe disease, and (b) no current diagnosis of schizophrenia, severe emotional disorder, or pervasive developmental disorders and (c) a full-scale IQ above 80. Moreover, as shown in Table 1, no significant group differences were observed in terms of gender ($X^{2}$(1) = 0.73, *p* = 0.39) and age (*t* =  − 1.36, *p* = 0.18) between ADHD and HC. Only marginal group difference was observed in hand ($X^{2}$(1) = 4.17, *p* = 0.04). The ADHD-C subtype also matched well with ADHD-I subtype in gender ($X^{2}$(1) = 0.34, *p* = 0.56), age (*t* = -0.05, *p* = 0.96), and hand ($X^{2}$(1) = 0.04, *p* = 0.98). The severity of symptoms was assessed by ADHD Rating Scale IV, which consists of 18 items matched from the DSM-IV creation, and includes dimensions of inattention score, hyperactivity/impulsivity score and total score [24].

**Table 1:** Demographic characteristics of the subjects in the present study

Demographics		HC	ADHD	*p*	ADHD-C	ADHD-I	*p*
Number		54	107		53	54	
Gender	M/F	40/14	84/21^*^	0.39	42/9^*^	42/12	0.56
Age (*y*)	Mean ± SD	11.6 ± 1.81	12.0 ± 1.71	0.18	11.6 ± 1.57	11.6 ± 2.02	0.96
Hand	L/R	0/53^#^	8/99	0.04	49/4	50/4	0.98
Inattention score	Mean ± SD	15.14 ± 3.70^†^	27.37 ± 3.06	< 0.001	27.58 ± 3.12	27.16 ± 2.99	0.49
Hyperactivity/impulsivity score	Mean ± SD	11.79 ± 2.48^†^	21.02 ± 6.46	< 0.001	24.60 ± 5.70	17.52 ± 5.10	< 0.001
Total score	Mean ± SD	26.93 ± 5.03^†^	48.40 ± 7.72	< 0.001	52.18 ± 7.02	44.69 ± 6.48	< 0.001

^*^Represents gender information for two patients was missing

^#^Indicates one healthy control was mix hand

^†^Represents only 14 HCs were recorded ADHD Rating Scale IV scores

### Data acquisition and preprocessing

Participants were instructed to sit in a dim lit room and keep their eyes closed for around 6 min. EEG data were obtained from EGI-128 channels (HydroCel Geodesic Sensor Net, Electrical Geodesics, Inc., Eugene, OR) with Net Station EEG Software. The impedance of all electrodes was kept below 50 kΩ during the data acquisition. All electrodes were physically referenced to Cz (fixed by the EEG acquisition system). The EEG recordings were amplified with a bandpass filter of 0.01–400 Hz (half-power cutoff) and digitized online at 1000 Hz.

Offline EEG processing was conducted using EEGLAB toolbox (https://sccn.ucsd.edu/eeglab/index.php) [25]. Thirty-eight lateral electrodes were excluded because of their susceptibility to movement interference, leaving 91 electrodes in the following analysis (see Figure S1). The resampling frequency was 250 Hz, and the bandpass filter band was 1–45 Hz. The signals were then re-referenced to the average reference. Electrodes containing excessive artifacts were manually checked and interpolated. The time series were subsequently inspected and curated prior to an independent component analysis (ICA) decomposition. ICA components associated with vertical and horizontal eye movements were visually identified and removed. The trimmed data were segmented into contiguous 2-s windows and any segments with voltages exceeding ± 100 µV were rejected, free of artifacts data were concatenated and the first 2 min were extracted for following analysis.

### Computing microstates features

The microstate analysis was performed using a Matlab plugin for the EEGLAB toolbox (http://www.thomaskoenig.ch/index.php/software/microstates-in-eeglab/). The current analysis was carried on Matlab R2018b [26]. Global field power (GFP) of the preprocessed resting-state EEG data was first determined at each time point for each subject. GFP is a measure of neuronal activity throughout the brain, which is calculated as the root of the mean of the squared potential differences at all electrodes from the mean of instantaneous potentials across electrodes as defined below:

1 $${\text{GFP}}\left( t \right) = \sqrt {\frac{{\mathop \sum \nolimits_{i = 1}^{n} \left( {V_{i} \left( t \right) - V_{{{\text{mean}}}} \left( t \right)} \right)^{2} }}{n}} ,$$

where $i$ is the electrode, $n$ represents the number of electrodes, $V$ represents measured voltage, $t$ is the time point.

Since scalp topographies remain quasi-stable around GFP peaks and present the highest signal-to-noise ratio, only EEG maps at the peaks were used for the subsequent clustering analysis. The selected GFP data were submitted to k-means clustering to identify the most dominant topographies as classes of microstates [20]. To further compare and interpret our results with previous studies, we also selected the most used four cluster numbers. The final maps were then quantified using global explained variance (GEV), which measures how well the spatial maps could explain the variance of the whole data. To reduce the influence of randomly selected initial template maps, we repeated the clustering procedure for hundreds of times and selected the microstates with the highest GEV [27]. The clustering analysis was first conducted at the individual level and then across subjects in each group. For cross-group comparison, we subsequently computed mean microstate topographies cross different groups and reoriented the group-mean maps as A–D according to their similarities to Koening et al.’s four-states normative microstate [28]. Each group-level map was then reoriented according to these group-mean topographies. The group-level spatial maps were further used as a reference map to back reconstruct information for each subject, where topographies at each time point were spatially correlated with each group-level map and labeled based on the most correlated map. Four microstate parameters for each subject were calculated: mean duration, time coverage, occurrence and transition probabilities [29]. The mean duration (in ms) is the average length of time a given microstate remains stable whenever it appears (yielding four features). The frequency of occurrence of each microstate is the average number of times per second that the microstate becomes dominant during the recording period (yielding four features). The coverage (in %) is the percentage of total recording time that the microstate is dominant (yielding four features). The transition probability quantifies the transformation from one state to another state (yielding 12 features). Altogether, a total of 24 features were achieved for subsequent analysis.

### Two-level statistical analysis on microstate features

To investigate whether the computed microstate parameters reflect variations between ADHD and HC, as well as different subtypes, we separated the analysis into two stages. At the first stage, we conducted group-level clustering analysis across all ADHDs and HCs, followed by calculating microstates parameters for each group. A two-way ANOVA with group and microstates as factors was then performed for each of the four computed microstate parameters to identify the significant microstate features, followed by two-sample *t* tests between patients and controls for each significant microstate feature. At the second stage, group-level clustering was conducted separately for ADHD-C and ADHD-I. We adopted the new topographies for back fitting and computed microstates parameters for each subtype participant. Then, we compared the group difference between ADHD-C and ADHD-I for each of the computed microstate parameters using ANOVA and two-sample *t* tests. Note that pairwise group comparison for all microstate parameters in each stage were corrected for multiple comparisons with Bonferroni correction with *p* < 0.05/24 comparisons.

### Independent component analysis on different frequency bands

The aim of this part was to objectively assess and compare the absolute EEG power between different groups. Spectral analysis of absolute power using FFT was carried out for the six frequency bands: delta (1–4 Hz), theta (4–8 Hz), low alpha (8–10 Hz), high alpha (10–12 Hz), beta (12–30 Hz), gamma (30–45 Hz), as well as TBR calculated by the ratio between theta and beta absolute power. The mean power of each frequency band in all channels of all ADHDs and HCs was organized into a *N*_subj_ × *N*_channel_ matrix (161 × 91) for each frequency band, obtaining seven power matrices. The formed power matrices were analyzed using ICA, which extracts maximally independent components (ICs) through maximization of entropy which measures uncertainty association with a random variable. Each component reflects coherent electroencephalogram variations within several-related channels across groups [30]. The approach reduces multiple comparisons required in channel-wise analyses and also dissects the brain into functionally independent networks [31]. The decomposed components were further tested for group differences between HCs and ADHDs using two-sample *t* tests, and the corresponding spatial components were converted to *Z* scores to select top contribution channels. As for comparison between two subtypes, seven power matrices were also similarly constructed on ADHD-C and ADHD-I, followed by ICA decomposition and group difference comparison. The ICA code is available for public use through the EEGIFT Toolbox (https://trendscenter.org/software/eegift) [32].

### Classification

**Fig. 1:**
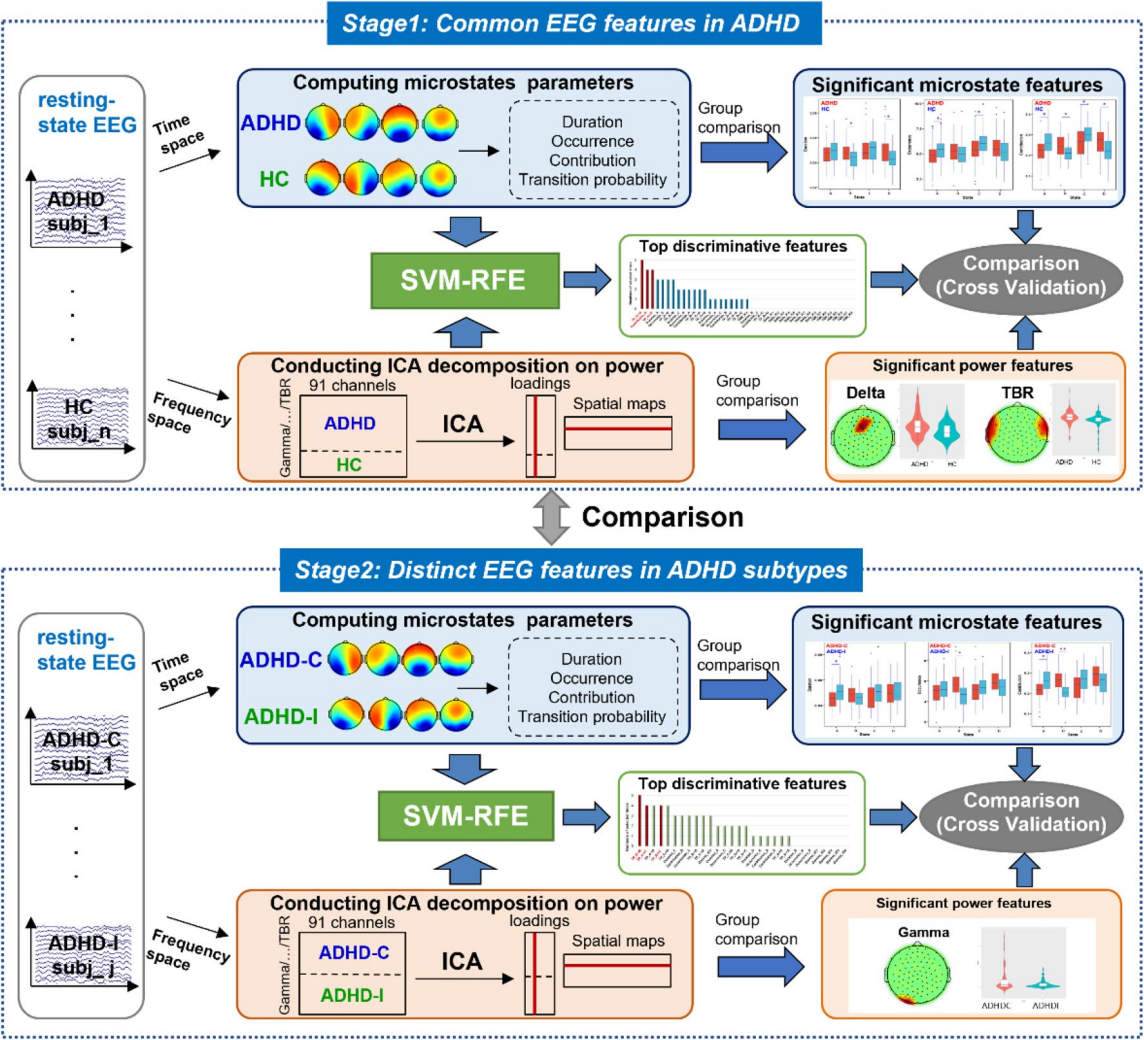
Flowchart of the study design

To provide further evidence for the effectiveness of the above significant features, we adopted a SVM with recursive feature elimination (SVM-RFE) algorithm [33] embedded in a balanced fivefold cross-validation framework. The SVM-RFE is able to determine a rank of *N* input features based on their importance in classification. One or more features having the smallest contribution are eliminated and the kernel matrix is updated using the remaining features. The process is repeated until a predetermined number of features remain [34]. In this study, we included all the microstate features and power features as original input features. We would like to compare whether the features identified from the above group differences also present significant discriminative power in classification (Fig. 1).

## Results

### Microstates between ADHD and HC

**Fig. 2:**
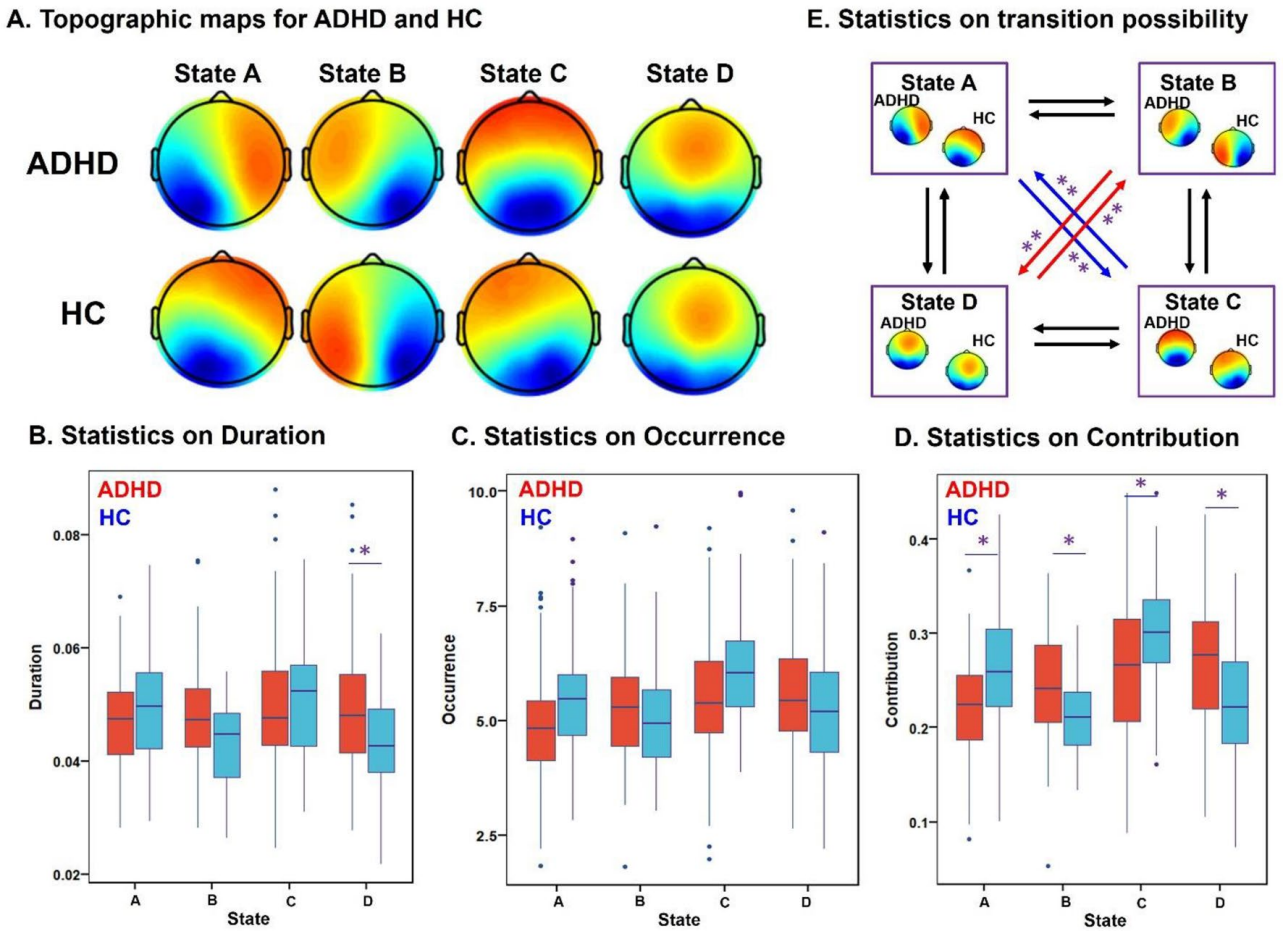
Topographic maps and statistical analysis from microstate features on ADHD and HC. **A** The four microstate maps consistently resembled those that were identified in the previous literature; **B**–**E**) post hoc pairwise group comparisons on four microstate features: mean duration (**B**), time coverage (**C**), occurrence (**D**), and transition probabilities (**E**). *Indicates 1.00e-5 < *p* < 2.08e-3. **Represents 1.00e-10 < *p* < 1.00e-5

The four microstates for patients and controls are presented in Fig. 2. In both groups, the microstate maps consistently resembled those that were identified in previous literature [16]: state A and state B with diagonal axis orientations of the topographic map filed, state C with anterior–posterior orientation and state D with a front-central location. The four microstates across participants explained 80.67% and 80.89% of the global variance in the patients and controls, respectively. The subsequent Kruskal–Wallis test showed no significant group differences between ADHDs and HCs for each topography map (*p*(A) = 0.99, *p*(B) = 0.98, *p*(C) = 0.93, *p*(D) = 0.99).

Based on the identified microstates, we computed four parameters for each subject: mean duration, time coverage, frequency of occurrence and transition probabilities. Two-way ANOVA analysis showed significant microstate × group interaction effects for mean duration (*F* = 8.54, *p* = 1.56e-5), time of coverage (*F* = 13.86, *p* = 1.10e-8), occurrence (*F* = 11.78, *p* < 1.00e-12), and transition probabilities (*F* = 12.07, *p* < 1.00e-12). Post hoc pairwise group comparisons (Fig. 2, Table S1) revealed that the contribution of state A (*p* = 3.39e-5) and state C (*p* = 1.46e-3) were markedly decreased in ADHD, while the contribution of state B (*p* = 2.07e-4) and state D (*p* = 1.26e-4) were significantly increased in ADHD compared to HC. These results are in keeping with the triple-network model of pathophysiology associated with ADHD [35], including aberrant salience-processing (state C) and frontoparietal network (state D). Moreover, the transition probability between state A and state C (*p* = 9.85e-7; *p* = 2.33e-7) was significantly decreased in patients, whereas increased between state B and D (*p* = 1.02e-7; *p* = 1.07e-6) in patients with ADHD compared to HC.

### Microstates patterns for ADHD-C and ADHD-I

**Fig. 3:**
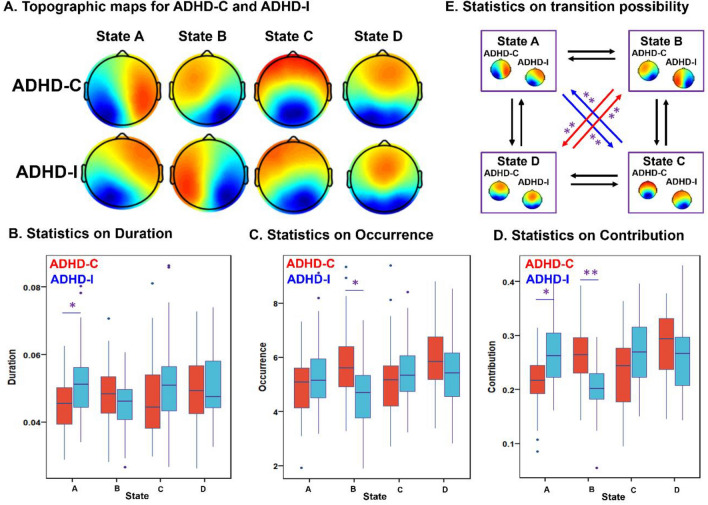
Topographic maps and statistic analysis from microstates features on ADHD-C and ADHD-I. **A** The four microstate maps consistently resembled those that were identified in the previous literature; **B**-**E**) post hoc pairwise group comparisons on four microstate features: mean duration (**B**), time coverage (**C**), occurrence, (**D**) and transition probabilities (**E**). *Indicates 1.00e-5 < *p* < 2.08e-3. **Represents 1.00e-10 < *p* < 1.00e-5

The four microstates for ADHD-C and ADHD-I are shown in Fig. 3. In both sub-groups, the four microstate maps markedly resembled those previously identified [16]. The four microstates across participants explained 80.06% and 80.31% of the global variance for ADHD-C and ADHD-I. The subsequent Kruskal–Wallis test showed non-significant group difference between two subtypes for each topography maps (*p*(A) = 0.99, *p*(B) = 0.95, *p*(C) = 0.95, *p*(D) = 0.99). Two-way ANOVA analysis based on the calculated four microstate parameters demonstrated significant microstate × group interaction effects for mean duration (*F* = 4.24, *p* = 5.90e-3), time of coverage (*F* = 8.77, *p* = 1.32e-5), occurrence (*F* = 6.47, *p* = 3.02e-4), and transition probabilities (*F* = 8.20, *p* < 1.00e-12). Not surprisingly, we found no significant differences in the general properties of microstates C and D between subtypes (Fig. 3, Table S2), as the common inattention characteristic featured by salience and frontoparietal network disruption was shared between the two subtypes. Instead, the occurrence and coverage of state B (associated with visual network) were increased in ADHD-C, while the duration and contribution of state A (associated with temporal network) were decreased in ADHD-C compared to ADHD-I. Furthermore, the transition probability between state A and state C (*p* = 9.25e-8; *p* = 1.78e-8) was decreased in ADHD-C, whereas increased from state B to state D (*p* = 9.86e-9; *p* = 2.54e-7) in patients with ADHD-C compared to ADHD-I.

### Frequency power analysis between different groups

**Fig. 4:**
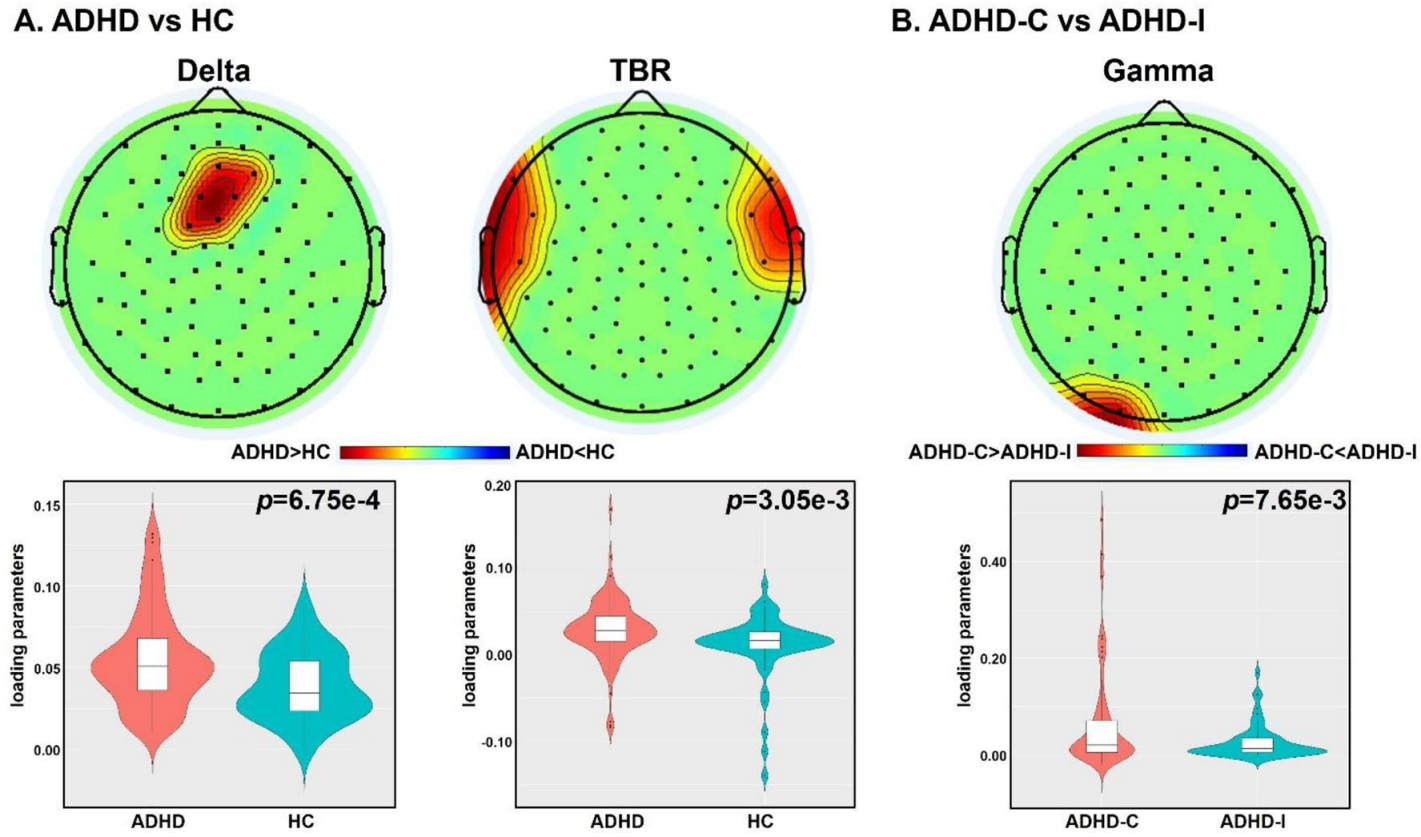
ICA analysis on power of different frequency bands between ADHD and HC (**A**) and subtypes (**B**). The upper panel represents the spatial map. The red region indicates higher contribution in HC than ADHD, as well as higher contribution in ADHD-C than ADHD-I. The lower panel represents the group difference of loading parameter of the selected component. The red and blue color separately represents HC and ADHD, as well as ADHD-C and ADHD-I

Each prepared power matrix was decomposed into 5, 6, 7, 8, 9, 10 components using ICA. Group difference between ADHD and HC were then assessed through two-sample *t* tests for each component. The components were selected if they passed Bonferroni correction with 0.05/component numbers and exhibited high activations within the channels. The corresponding spatial maps of the selected components were converted to *Z* scores to select the top contributing channels with |Z|> 2.As shown in Fig. 4A, one delta component (*p* = 6.75e-4, passed Bonferroni correction with 0.05/9) and one TBR component (*p* = 3.05e-3, passed Bonferroni correction with 0.05/8) showed significant group differences between ADHD and HC. Since spatial map has been adjusted as HC > ADHD, ADHD-C > ADHD-I on the mean of loading parameters, then the red region indicates higher contribution in HC than ADHD, as well as higher contribution in ADHD-C than ADHD-I. Higher power in ADHD compared to HC was observed in the delta component spanning in the fronto-central area and the TBR component located in the bilateral fronto-temporal area. We did not reveal any significant group differences in other power matrices.

When comparing group differences between ADHD-C and ADHD-I, we found that only one gamma power in the posterior occipital region was significantly increased (*p* = 7.65e-3, passed Bonferroni correction with 0.05/6) in ADHD-C compared ADHD-I (Fig. 4B). No significant group differences were observed in other frequency bands.

### Classification

**Fig. 5:**
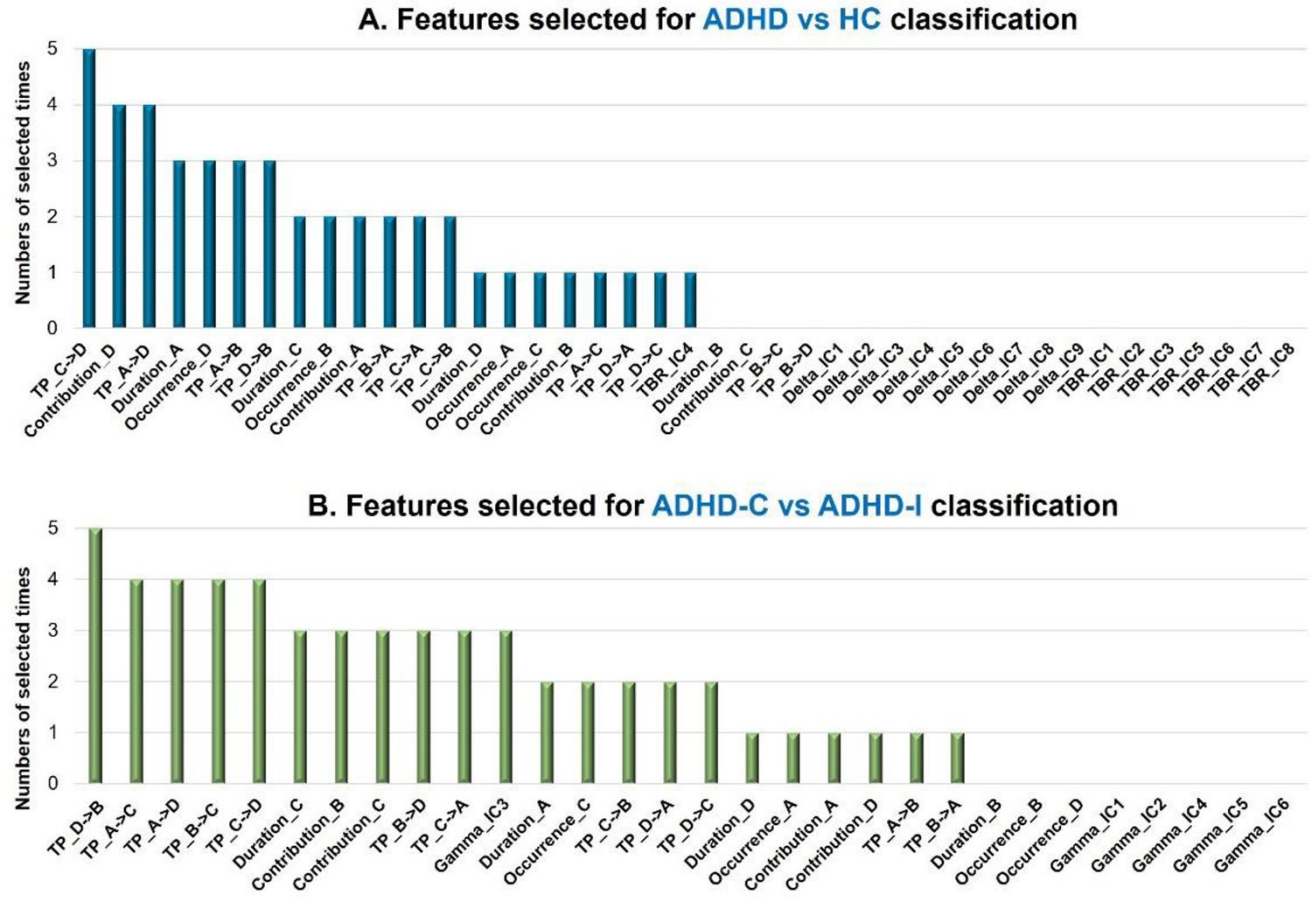
Comparison between the selected features of SVM-RFE classification on ADHD vs HC (**A**) and two subtypes (**B**). **A** Indicates the ranking of all features when discriminating ADHD from HC. **B** Indicates the ranking of all features on the classification of ADHD-C and ADHD-I

We fed all the microstate features, delta and TBR power components into the SVM-RFE model for ADHD and HC classification. Figure 5 indicates the sum of times of discriminative features selected in all the fivefold cross-validation test. The best accuracy, sensitivity and specificity for discriminating ADHD from HC were 72.7%, 66.7%, and 75.7%, respectively. The most frequently selected feature was transition probability from state C to state D, followed by the contribution of state D and the transition probability between sate A and state D (Fig. 5A). However, little power features were selected in the top discriminative feature lists. When discriminating two subtypes, all the microstate features and gamma power components were used as the input features. The best accuracy, sensitivity and specificity for discriminating two subtypes were 73.8%, 74.1%, and 73.6%, respectively. As shown in Fig. 5B, the most frequently selected feature was the transition probability from state D to state B, followed by the transition probability from state A to state C, state A to state D, state B to state D and state C to state D. Compared to microstate features, IC3 of gamma power which activated in the posterior occipital region was selected in three out of five tests.

## Discussion

In this work, we adopted temporal microstate dynamics and spectral power features to analyze group differences between ADHD and HC, as well as ADHD subtypes. Results demonstrated that (1) both subtypes showed shared aberrant patterns on dynamics of salience (state C) and frontal-parietal network (state D), as well as higher delta power in the fronto-central area and higher TBR power in the fronto-temporal area for ADHD compared to HC. (2) Remarkable subtype microstate differences were found primarily in the visual network (state B) between ADHD-I and ADHD-C as well as higher gamma signals over the posterior occipital region in ADHD-C compared to ADHD-I. (3) The top discriminative features selected from SVM-RFE model well replicated the results identified from group differences, which achieved an accuracy of 72.7% and 73.8% separately in classifying ADHD patients and two subtypes.

### Shared patterns in ADHD-C and ADHD-I

Compared to HC, we showed that the occurrence and coverage of state C were decreased in ADHD (*p* = 0.002; *p* = 0.0015), while the duration and contribution of state D were observably increased (*p* = 0.0016; *p* = 0.0001) compared to HC. These two states were also highlighted in the subsequent selected features of SVM-RFE model. On the one hand, microstate state C has been attributed to the salience network [21], comprising anterior insular and anterior cingulate cortex, which has a central role in the detection of behaviorally relevant stimuli and the coordination of neural resources [36]. There is a wealth of neuroimaging evidence for ADHD-related abnormalities in the structure and function of this network [37]. For example, a meta-analytic comparison showed that patients with ADHD presented decreased GM volume in salience network [38]. Moreover, a previous study using resting-state functional magnetic resonance imaging scans of 19 drug-naïve boy with ADHD and 23 controls indicated that children with ADHD presented a decreased anti-correlation between the dorsal anterior cingulate cortex and the default mode network [39]. During a Go/NoGo fMRI task, the evoked dorsal anterior cingulate cortex-ventrolateral prefrontal cortex in the salience network was positively correlated with NoGo accuracy, and negatively correlated with severity of inattention symptoms [40]. The decreased activation on salience network would help us understand the inattention symptoms in ADHD patients, which prevented the children from encoding the stimuli effectively and leading to general performance deficits [41]. Another study further suggested that the brain-computer-interface (BCI)-based attention training facilitated attention improvement in children with ADHD primarily through renormalizing salience network processing [42]. On the other hand, class D is correlated with frontal and parietal cortex function (frontoparietal network), also known as the executive control circuit, which is involved in sustained attention, inhibition, work memory and goal-directed decision making [43]. The dysregulation of frontoparietal network (FPN) systems has been increasingly reported in ADHD. Cai et al. revealed that the connectivity between right dorsolateral prefrontal cortex and posterior parietal cortex in the frontoparietal “central executive” network emerged as the most distinguishing link to distinguish the ADHD and HC [40]. Children with ADHD have higher fractional anisotropy values in white matter in the right frontal regions [44]. In addition to the executive deficits, the abnormal increased class D state might be associated with emotion dysregulation and impulsivity in ADHD, which was similar to the FPN hyperconnectivity for ADHD in the pooled rs-fMRI meta-analyses [35], as the abnormal emotional regulation would induce an excessive activation of FPN[45].

In parallel, power analysis revealed that higher delta power in the fronto-central area and higher TBR power in the fronto-temporal area were revealed in ADHD compared to HC, which were supported by many previous studies [46–49]. For example, children with ADHD have been revealed increased power in the slow EEG frequency bands, including delta and theta bands over centro-parietal regions [46, 47]. Accumulating evidences confirm that TBR is closely related to ADHD, as patients with higher TBR showed more severe inattentive issues [50, 51]. Moreover, frontal TBR is negatively related to executive, most notably attentional control [48, 49], and elevated TBR in ADHD was related to more difficulty in inhibiting surrounding stimuli [52].

### Distinct patterns in ADHD-C and ADHD-I

Besides the shared abnormal patterns in ADHD-C and ADHD-I, the distinct patterns are valuable for understanding the mechanisms of ADHD symptom dimensions. Because the distribution of ADHD subtypes evolves with individual development and functional differentiation, and the mechanisms of their evolution remain unknown [2, 3]. The differential patterns of ADHD-I and ADHD-C may reflect stage-specific brain function properties of the two subtypes. When comparing the differences between subtypes, ADHD-C presented higher occurrence and coverage of state B than ADHD-I (*p* = 9.35e-5; *p* = 1.51e-8). In the subsequent SVM-RFE analysis, state B was also revealed as the top discriminative feature between two subtypes. State B has been attributed to the visual network [21]. In a Go/NoGo task of inhibitory control, children with ADHD-C activated bilateral medial occipital lobe to a greater extent than children with ADHD-I [53]. Consistent with temporal microstate results, when comparing group differences of frequency power between ADHD-C and ADHD-I, we also identified higher gamma power in the posterior occipital region of ADHD-C compared ADHD-I. Gamma-band responses (GBR) from visual cortex play a pivotal role in visual processing [54, 55]. The hyper-activated occipital gamma and state B might suggest ADHD-C exhibited a stronger visual functional activity compared to children with ADHD-I. The over-activation of visual function is widely reported in patients with ADHD during cognitive tasks [56], which generally improved the task performance and might represent a compensation provided by the visual process [57–59]. This stronger visual abnormality in ADHD-C may reflect an underlying mechanism of subtype differences. Previous studies have found that visual abnormalities in ADHD are developmentally specific for age and present only at specific stages of cognitive development [60], which may also suggest a stage-specific emergence of subtype differentiation which may be less pronounced with the maturation of cognitive development. And some studies have also suggested that excessive activation of the visual cortex may be a sign of insufficient inhibition control [61]. A task-based experiment indicated that the unusual evoked GBRs of ADHD patients brought a lack of early memory-based classification which possibly resulted in an impaired ability to rapidly reallocate attentional resources to relevant stimuli [62]. Our findings might suggest children with ADHD-C show less control on themselves than ADHD-I, who exhibited more visual activation when asked to close eyes. Moreover, because of the declined top-down eye movement control ability of ADHD [63], children with ADHD-C might exhibit impulsivity of opening their eyes in an eye-closed experiment, leading to hyper-activated visual network.

Our study has several limitations. First, our sample size was limited, and we only have single site data which containing ADHD and HC, therefore, we cannot replicate the classification study because of the lack of another independent dataset. However, we have tried our best to reduce the over-fitting and improve the stability of the results [64–66], namely, when conducting SVM-RFE analysis, we used a nested fivefold cross-validation. Second, marginal group difference was observed in handedness between ADHD and HC, which may leave possible effect on the results. However, the effect of handedness on ADHD is controversial. Some empirical studies have found evidence for a higher prevalence of atypical handedness in individuals with ADHD compared to neurotypical individuals [67, 68]. While other studies failed to establish such an association [69, 70]. Overall speaking, this work suggests EEG microstates could be a potential imaging biomarker for ADHD identification or subtype discrimination. In the future work, we would like to include more longitudinal data to predict the treatment outcome of subtypes, which would be an important extend for the current study.

### Supplementary Information

Below is the link to the electronic supplementary material.

Supplementary file1 (DOCX 629 KB)
