# Supplementary material for: Aberrant brain dynamics and spectral power in children with ADHD and its subtypes
Source: Eur Child Adolesc Psychiatry. 2022 Aug 22;32(11):2223–34. doi: 10.1007/s00787-022-02068-6 (PMC10576687; doi:10.1007/s00787-022-02068-6)
Supplement: Supplementary file 1 — Supplementary file1 (DOCX 629 KB) [file 787_2022_2068_MOESM1_ESM.docx]

**Supplementary material**

## Supplementary tables

**Table S1. Two sample t-test for each microstate feature between ADHD and HC**

| **Parameters** | ***p* value** | **Transition probability** | ***p* value** |
| --- | --- | --- | --- |
| Duration (A) | 0.07 | state A🡪state B | 0.85 |
| Duration (B) | 2.93E-03 | **state A🡪 state C** | **9.85E-07*** |
| Duration (C) | 0.59 | state A🡪 state D | 0.71 |
| **Duration (D)** | **1.57E-03*** | state B🡪 state A | 0.97 |
| Occurrence (A) | 5.46E-03 | state B🡪 state C | 0.34 |
| Occurrence (B) | 0.31 | **state B🡪 state D** | **1.02E-07*** |
| Occurrence (C) | 2.75E-03 | **state C🡪 state A** | **2.33E-07*** |
| Occurrence (D) | 0.12 | state C🡪 state B | 0.52 |
| **Contribution (A)** | **3.39E-05*** | state C🡪 state D | 0.95 |
| **Contribution (B)** | **2.07E-04*** | state D🡪 state A | 0.42 |
| **Contribution (C)** | **1.46E-03*** | **state D🡪 state B** | **1.07E-6*** |
| **Contribution (D)** | **1.26E-04*** | state D🡪 state C | 0.96 |

**Table S2. Two sample t-test for each microstate feature between two subtypes**

| **Parameters** | ***p* value** | **Transition probability** | ***p* value** |
| --- | --- | --- | --- |
| **Duration (A)** | **3.89E-04*** | state A🡪 state B | 0.27 |
| Duration (B) | 0.03 | **state A🡪 state C** | **9.25E-08*** |
| Duration (C) | 0.07 | state A🡪 state D | 0.15 |
| Duration (D) | 0.79 | state B🡪 state A | 0.19 |
| Occurrence (A) | 0.24 | state B🡪 state C | 0.25 |
| **Occurrence (B)** | **9.35E-05*** | **state B🡪 state D** | **9.86E-09*** |
| Occurrence (C) | 0.25 | **state C🡪 state A** | **1.78E-08*** |
| Occurrence (D) | 0.06 | state C🡪 state B | 0.08 |
| **Contribution (A)** | **1.70E-05*** | state C🡪 state D | 0.07 |
| **Contribution (B)** | **1.51E-08*** | state D🡪 state A | 0.14 |
| Contribution (C) | 0.01 | **state D🡪 state B** | **2.54E-07*** |
| Contribution (D) | 0.18 | state D🡪 state C | 0.12 |

## Supplementary figures


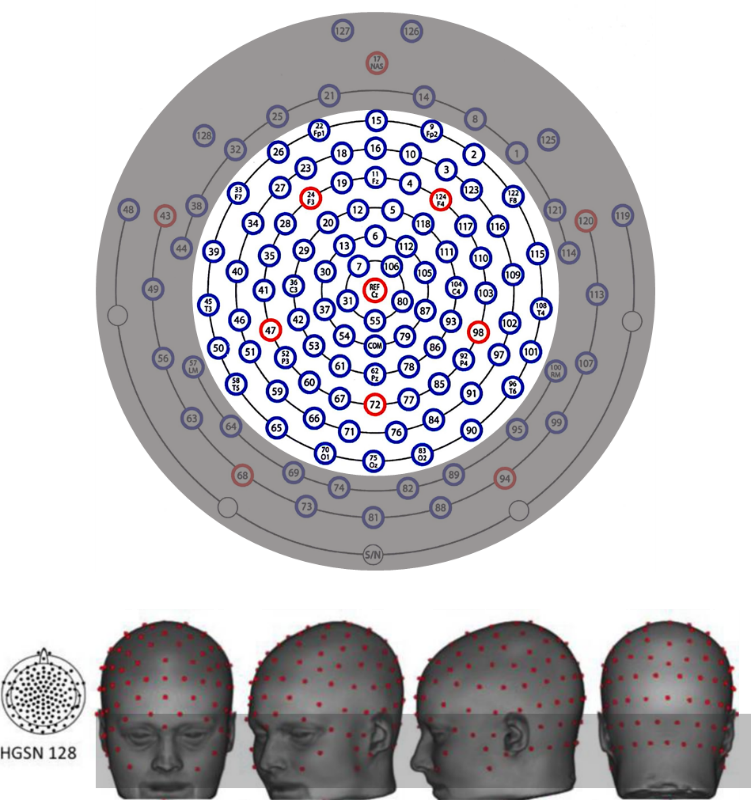


**Figure S1.** Electrodes used to analysis. The shaded areas are 38 peripheral electrodes that are not included in the data analysis (3D head models refers to (1)).

**References**

1. Richards JE, Boswell C, Stevens M, Vendemia JM (2015) Evaluating methods for constructing average high-density electrode positions. Brain Topogr 28(1):70-86.
